# Complex Dermatological Manifestations of Poorly Controlled Diabetes: A Case of Acquired Ichthyosis

**DOI:** 10.1155/crdm/4532886

**Published:** 2026-01-27

**Authors:** Sam Fathizadeh, Alexander D. Woods, Saul Turcios Escobar, Maria Tsoukas

**Affiliations:** ^1^ College of Medicine, University of Illinois Chicago, Chicago, Illinois, USA, uic.edu; ^2^ Department of Dermatology, University of Illinois Chicago, Chicago, Illinois, USA, uic.edu; ^3^ Department of Pathology, University of Illinois Chicago, Chicago, Illinois, USA, uic.edu

**Keywords:** acquired ichthyosis, case report, dermatologic manifestations, diabetes mellitus, ichthyosis

## Abstract

Acquired ichthyosis (AI) is a rare dermatological disorder characterized by dry, scaly skin. This case involves a 67‐year‐old Hispanic male with poorly controlled diabetes mellitus (DM) who presented with generalized dryness and itchiness after diabetic ketoacidosis. Examination revealed polygonal scales with erythema, and biopsy confirmed AI. Laboratory tests showed elevated glucose, dyslipidemia, hyponatremia, hyperkalemia, and Stage IIIb chronic kidney disease. Treatment included moisturizers, antihistamines, antifungal shampoo, topical corticosteroids, tacrolimus, and optimized DM management, leading to improvement. AI is often linked to systemic conditions like malignancy, autoimmune diseases, infections, and certain medications. Diagnosis is clinical and biopsy‐supported, requiring a systemic workup to identify underlying causes. Poorly controlled DM was significant in this case, highlighting the importance of comprehensive assessment. Early recognition and understanding of AI’s association with DM can optimize treatment and reduce morbidity.

## 1. Introduction

Acquired ichthyosis (AI) is a rare dermatological disorder characterized by dry, rough, and scaly skin. The condition can be associated with underlying causes, including systemic diseases such as hypothyroidism, malignancy, and certain medications [1, 2]. Currently, AI associated with diabetes mellitus (DM) has been reported in two cases in younger patients [3, 4]. Here, we report on an older patient with poorly controlled DM who developed AI.

This case is unique as it represents one of the few reports of AI in an older adult with poorly controlled DM. Prior cases have been limited to younger patients, and our report expands the demographic spectrum, supporting the importance of glycemic control as a potential modifiable factor in disease resolution [3–6].

## 2. Case Presentation

A 67‐year‐old Hispanic male with uncontrolled DM presented with a five‐week history of generalized dryness and itchiness, initially localized to his back and subsequently spreading throughout his body. Of note, the patient was recently hospitalized for diabetic ketoacidosis. The episode was managed with intravenous insulin and fluids during hospitalization, resulting in clinical stabilization prior to dermatologic evaluation. He also reported a long‐standing history of heavy alcohol consumption and a 50‐pack‐year smoking history.

Dermatological examination revealed dry polygonal scales with interscale erythema over his legs, scalp, arms, back, and abdomen (Figures 1(a) and 1(b)). Histopathologic examination of specimens from the left shoulder showed epidermal atrophy, compact orthokeratosis, hypogranulosis, focal parakeratosis, mild spongiosis, and superficial perivascular lymphocytic inflammation, including numerous eosinophils, confirming the diagnosis of AI (Figure 2). The left shoulder was selected for biopsy because it exhibited representative scaling morphology without excoriation, allowing optimal histologic sampling. Although this region was not photographed separately, the clinical appearance was similar to the scaling observed on other affected sites, including the legs and back (Figures 1(a) and 1(b)).

**Figure FIGURE 1:**
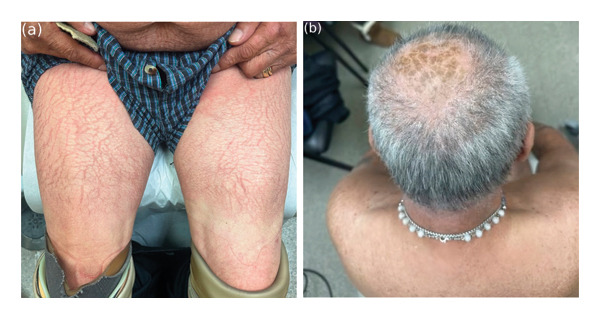
Irregularly arranged polygonal scales of varying thicknesses and interscale erythema over the anterior thighs (a) and scalp (b).

**Figure FIGURE 2:**
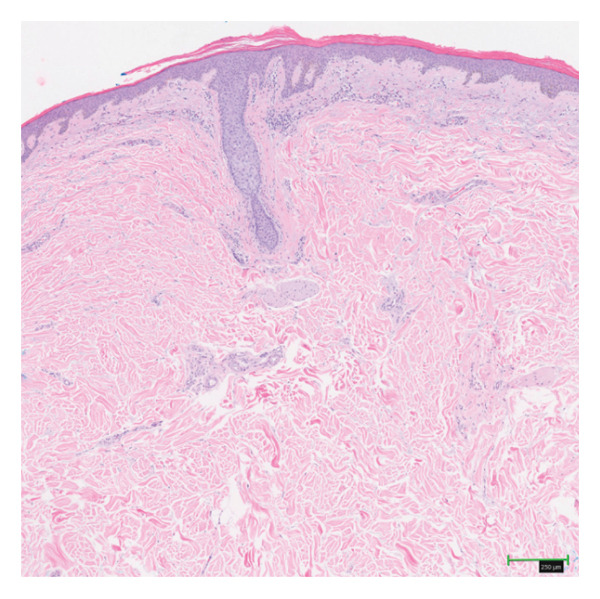
Epidermal atrophy, compact orthokeratosis, hypogranulosis, focal parakeratosis, mild spongiosis, and superficial perivascular lymphocytic inflammation, including numerous eosinophils (H & E, 50x).

Given the association of AI with underlying conditions and malignancy, a complete blood count, comprehensive metabolic panel, and lipid panel were ordered, and age‐appropriate screening was recommended [1, 2]. Laboratory findings included elevated fasting plasma glucose (411 mg/dL), dyslipidemia, hyponatremia, hyperkalemia, chronic kidney disease Stage IIIb (creatinine 1.40 mg/dL), and normal liver function tests (Table 1); hemoglobin A1c was not obtained at initial presentation. The patient was advised on daily moisturization with a petrolatum‐based emollient and prescribed loratadine 10 mg once daily for pruritus, ketoconazole 2% shampoo twice weekly for scalp scaling, triamcinolone 0.1% cream twice daily to affected areas for two weeks, and tacrolimus 0.1% ointment twice daily for facial involvement. These were selected to address xerosis, inflammation, and confounding seborrheic dermatitis, all contributing to pruritus. The patient was advised to continue close follow‐up with his primary care physician, who was working to control his DM with insulin, sitagliptin, and metformin as well as comorbid conditions with atorvastatin and hydrochlorothiazide.

**Table TABLE 1:** Laboratory results at presentation and six‐month follow‐up.

Test	Result	Reference range	Units	Timeline
Fasting plasma glucose	411	70–100	mg/dL	At presentation
Fasting plasma glucose	144	70–100	mg/dL	6‐month follow‐up
Hemoglobin A1c	6	4.0–5.7	%	6‐month follow‐up
Creatinine	1.4	0.6–1.3	mg/dL	At presentation
Creatinine	1.07	0.6–1.3	mg/dL	6‐month follow‐up
Sodium	129	135–145	mmol/L	At presentation
Sodium	133	135–145	mmol/L	6‐month follow‐up
Potassium	5.6	3.5–5.1	mmol/L	At presentation
Potassium	4.6	3.5–5.1	mmol/L	6‐month follow‐up
LDL‐C	156	< 130	mg/dL	At presentation

Upon initiation of aggressive topical therapy, the patient noticed skin improvement. At 6 months, the patient’s exam revealed resolution of dryness, scaling, and erythema (Figures 3(a) and 3(b)), which was further maintained on emollients only. Although an initial hemoglobin A1c was not obtained, his point‐of‐care glucose was markedly elevated at presentation. At the six‐month follow‐up, his hemoglobin A1c level was 6.0%, and his fasting plasma glucose had improved from 411 mg/dL at presentation to 144 mg/dL (Table 1), reflecting markedly better glycemic control following his prior episode of diabetic ketoacidosis.

**Figure FIGURE 3:**
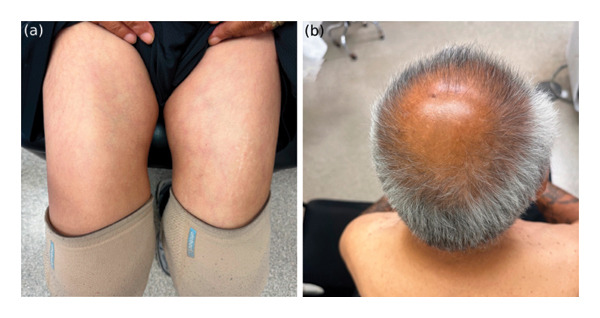
Six‐month follow‐up showing resolution of ichthyosis with improvement in skin texture and reduction of erythema over the anterior thighs (a) and scalp (b).

## 3. Discussion

DM is a metabolic disorder associated with various cutaneous manifestations. Common skin presentations in individuals with DM include diabetic dermopathy, necrobiosis lipoidica diabeticorum, impaired wound healing, and diabetic foot ulcers [7]. Other skin complications of DM include eruptive xanthomatosis, acanthosis nigricans, and candidal infections [7]. Our patient did not exhibit these DM skin findings but presented with AI, characterized by generalized scaling and dryness, prompting an investigation into potential underlying systemic conditions.

Unlike congenital forms of ichthyosis, which are present from birth, AI typically manifests later in life, often in adulthood. The exact cause of AI is not fully understood, but it often arises secondary to systemic conditions or external factors. AI has been described as a paraneoplastic phenomenon associated with malignancy (most often Hodgkin or non‐Hodgkin lymphoma, but also breast, lung, liver, and bladder cancer), and it can also correlate with autoimmune conditions, chronic infections (e.g., human immunodeficiency virus, hepatitis C, and tuberculosis), and certain medications (e.g., cimetidine, clofazimine, hydroxyurea, and nicotinic acid) [1, 2]. In addition, hypothyroidism, chronic renal failure, and nutritional deficiencies (e.g., vitamin A deficiency) are among the potential triggers associated with AI [1, 2]. In some cases, an underlying cause of AI cannot be identified. Although our patient had Stage IIIb chronic kidney disease, renal function improved with glycemic control and hydration, and the temporal association of disease onset with diabetic ketoacidosis rather than uremia suggests that CKD was unlikely to be the primary driver of the cutaneous findings.

AI often presents as scaling, particularly on the extensor aspects of the lower extremities, sparing flexural creases, and may involve the trunk and scalp [1, 2]. There may be associated pruritus and burning sensation, along with diffuse alopecia, leading to discomfort and cosmetic concerns for affected individuals [1, 2]. Diagnosis is clinical and can be supported with a biopsy. Upon diagnosis, it is essential to investigate for any underlying causes.

The mainstay of treatment for AI involves addressing the underlying systemic condition or inciting medication. Treatment of the underlying condition often leads to improvement in the ichthyosis. In addition, symptomatic treatment of AI involves moisturizers, emollients, and keratolytics to hydrate the skin, alongside alpha‐hydroxy acids (e.g., lactic or glycolic acid) or topical retinoids to promote skin cell turnover [1, 2].

Our case highlights an association between AI and DM in an older patient. The temporal association of uncontrolled DM with AI onset and improvement in symptoms with glycemic control and topical treatments support this association. Our patient, a 67‐year‐old man, presents a distinct demographic, as previous cases have been reported only with younger patients. While DM is linked with several cutaneous disorders, cases of AI in patients with DM are sparse, particularly involving an older demographic. Current literature highlights two cases: Sanli et al. presented a case of AI in a 20‐year‐old man that resolved with the initiation of insulin therapy, and Scheinfeld et al. discussed a case of new‐onset ichthyosis in a 14‐year‐old diabetic [3, 4]. Yosipovitch et al. further discussed varying degrees of ichthyosiform changes on the shins of young adult patients (48% of patients) with DM, and Pavlovic et al. found AI is the most common skin finding among young patients with DM (22% of patients) [5, 7]. However, both studies may represent asteatotic eczema, as it is unclear what diagnostic criterion was used, and these patients did not undergo histopathologic evaluation.

The mechanism underlying these skin changes remains incompletely understood, but the association between AI and DM is potentially linked to advanced glycosylation in structural proteins [5]. Glycosylated skin products are believed to disrupt normal skin barrier development, leading to dysfunctional collagen and reduced skin elasticity and resilience [4]. Collagen cross‐linking and glycosylation in the skin have previously been observed to correlate with hemoglobin A1c levels, a marker of hemoglobin glycosylation that reflects prolonged exposure to high blood glucose levels [3]. This association may demonstrate the relationship between glycemic control and skin health [3]. The prognosis of DM‐associated AI, and whether the disease course consistently improves with a reduction in A1c, remains to be fully elucidated.

In our patient’s case, poorly controlled DM stands out as a significant contributing factor to the development of AI, supporting the importance of comprehensive assessment in such scenarios. Our case furthers the understanding of AI as a potential dermatological manifestation in older adults with DM. Knowledge of this association and early recognition can help reduce the associated morbidity of both conditions. Additional research into the mechanisms of this association, as well as whether it improves significantly with glycemic control and optimization of therapeutic approaches, is warranted.

## Funding

No funding was received for this manuscript.

## Ethics Statement

This study was conducted in compliance with relevant ethical guidelines, including the Declaration of Helsinki.

## Consent

The patient in this manuscript has given written informed consent to publication of their case details.

## Conflicts of Interest

The authors declare no conflicts of interest.

## Data Availability

All data supporting the findings of this case are available from the corresponding author upon reasonable request, in accordance with the journal’s Data Protection and Privacy Policy.
